# Pulvinar sign and abnormally high CSF WBC in posterior reversible encephalopathy syndrome

**DOI:** 10.1002/ccr3.3219

**Published:** 2020-08-19

**Authors:** Whitney Kagabo, Kamran Imam, Antonio Liu

**Affiliations:** ^1^ Loma Linda University School of Medicine Loma Linda California; ^2^ Department of Neurology Adventist Health White Memorial Los Angeles California

**Keywords:** Atypical PRES, abnormal CSF pleocytosis, pulvinar sign

## Abstract

Posterior reversible encephalopathy syndrome (PRES) is a reversible disease process in which cerebral edema is secondary to a trigger causes neurological symptoms. Our case presents an unusual MRI location and CSF analysis in a patient with PRES.

## Graphical abstract

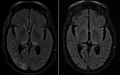

## 1 INTRODUCTION

Posterior reversible encephalopathy syndrome (PRES) is a neurological disease presenting with an array of clinical symptoms and concomitant magnetic resonance imaging (MRI) findings of cerebral edema. We present a patient who has two atypical features, pulvinar involvement and abnormally high cerebrospinal fluid (CSF) white count. Both features are seldom described in PRES.

Posterior reversible encephalopathy syndrome (PRES) is an acute neurological syndrome diagnosed via clinical and radiological presentations. PRES presents with headaches, altered mental status, seizures, and/or visual disturbances. Magnetic resonance imaging (MRI) demonstrates reversible subcortical vasogenic edema.^1^ The mechanism is unclear but believed to be due to a combination of endothelial injury, vasospasm/vasoconstriction, and failure of cerebral autoregulation.^2^ It is associated with other medical conditions such as hypertension, renal failure, eclampsia, immunosuppressant therapy, and autoimmune disorders. PRES is commonly triggered by hypertensive emergencies with blood pressures (BP) ranging from 160‐268/60‐144 mm Hg.^3^ The most important step in managing PRES is treating the initiating factor.

## 2 CASE PRESENTATION

A 67‐year‐old, right‐handed female patient, presented to our emergency department (ED) for altered mental status. She had previously been at an outside clinic earlier that day for an evaluation of chronic epigastric pain radiating to her back. She received a Toradol shot and felt tired afterward. Her condition worsened and she was brought into the ED where she was found to be obtunded and was intubated for airway protection. Neurologic examination was very limited secondary to sedation. Per family, the patient had a 2‐week history of malaise, generalized weakness, headache, incontinence, and eventually altered mental status. She had previously been healthy and independently performing all activities of daily living. She could drive and took care of her grandchildren.

**Figure 1:**
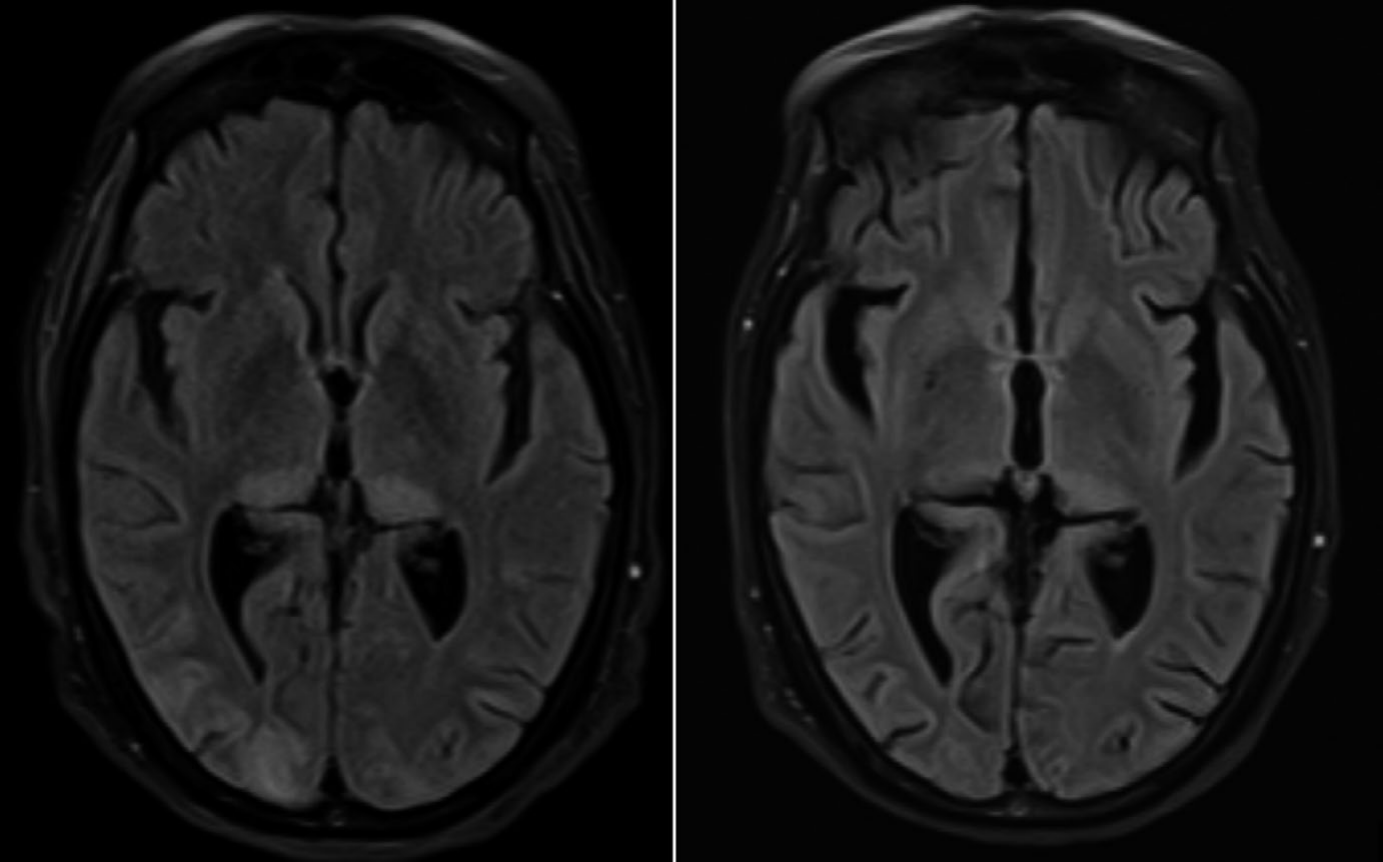
Fluid‐attenuated inversion recovery of MRI on day 2 of admission showing abnormal signal at bilateral pulvinar areas. On day 10 of admission, repeat MRI shows resolution. Also noted is the typical involvement of right occipital cortical and subcortical white matter in PRES

On examination, her vital signs included temperature: 98.3°F, pulse: 99/min, BP: 201/94 mm Hg, and oxygen saturation: 98% after intubation. Initial laboratory work demonstrated a normal complete blood count (CBC) and normal coagulation. Blood chemistry was notable for an elevated calcium of 14.5 mg/dL. Liver function tests demonstrated an elevated alkaline phosphatase (ALP) of 208 U/L; renal function laboratories were within normal limits. Cerebral spinal fluid (CSF) analysis showed a white blood cell count (WBC) of 91 with a lymphocyte value of 90, and a protein of 32. CSF testing for herpes simplex virus, coccidioidomycosis, cryptococcus, West Nile virus, and syphilis were all negative. Head computer tomography (CT) and magnetic resonance venography (MRV) were negative. Electroencephalography (EEG) repeatedly showed generalized slowing. On day 2, MRI fluid‐attenuated inversion recovery (FLAIR) showed abnormal posterior subcortical signal density with bilateral thalamic pulvinar involvement (Figure 1).

The patient was admitted to the intensive care unit (ICU) and underwent aggressive blood pressure management. Her blood pressure was brought down to 140/57 mm Hg over 4 days. Her mentation slowly improved over 1 week; she was extubated on day 8. Repeat blood work showed a normal chemistry with a calcium of 7.4 mg/dL. Her ALP had decreased to 125 U/L. Her brain was followed with serial MRIs. By day 10, her FLAIR abnormality had almost resolved. Repeat EEG showed delta activity. A repeat CSF analysis showed a WBC of 2 and a protein of 24. NMDA receptor antibody was negative both in serum and CSF. Prion study was negative with negative RT‐QuIC and no 14‐3‐3 protein.

Systemic workup demonstrated pancreatic cancer. The patient neurologically continued to improve to a close to baseline state. She was discharged home after a 20‐day stay in the hospital. A 1‐month postdischarge phone call revealed the patient had remained at her baseline mentation. Unfortunately, the patient eventually passed away from her pancreatic cancer 5 months later.

## 3 DISCUSSION

Posterior reversible encephalopathy syndrome (PRES) is a neurological disease presenting with an array of clinical symptoms such as headaches, vision changes, seizures, and MRI findings of cerebral edema typically in the posterior cerebrum. The most prevalent regions of presentation according to the McKinney study demonstrated the involvement of the parietooccipital region 98.7%, posterior frontal 78.9%, and temporal 68.4% regions.^4^ In this case, there were two unusual aspects to the patient presentation, the location of the edema and the CSF analysis findings.

This patient presented with cerebral edema located in the posterior cortical region and pulvinar region. This is particularly unique not only because it is such an atypical location for PRES but also because it is a very specific location for a different disease, variant Creutzfeldt‐Jakob disease (vCJD). Pulvinar sign refers to the bilateral symmetrical hyperintensities in the posterior nucleus of the thalamus, and it is detected using T2‐weighted or FLAIR MRI.^5^ It is reported to have a sensitivity of 78%‐90% and a specificity of 100% for vCJD in the right clinical setting.^6^ Variant Creutzfeldt‐Jakob disease presents with a constellation of symptoms, predominantly: ataxia, psychiatric/behavioral symptoms, painful dysesthesia, involuntary movements, and cognitive impairment.^7^ It is a relentlessly progressive and uniformly fatal disease. This is strikingly different than the presentation of typical CJD whose presentation is dominated by decompensating dementia. This case demonstrated the importance of differentiating PRES from vCJD as it had mixed MRI findings of PRES and vCJD but clinical findings suggestive of PRES.

The second unusual aspect of our patient's presentation of PRES was an elevated CSF WBC. Even though a lumbar puncture is not required to diagnose PRES, many patients undergo a lumbar puncture to rule out other diagnoses. In these cases, common CSF findings are protein elevation. The Ellis study demonstrated that protein elevation was associated with the severity of PRES, with higher elevations of CSF protein being associated with increased severity of vasogenic edema.^3^ High CSF WBC has been rarely described in PRES. Multiple studies relating to CSF findings in PRES did not find a significant elevation in WBC with a median WBC count of 1,^3^ whereas our patient presented with a CSF WBC of 91 with lymphocyte predominance. It is important to document this, as to not confuse future presentations with encephalitis. The cause of the elevated CSF WBC is not well understood, and it may point toward inflammation being part of PRES; more data are needed in order to further explain the observation.

This case illustrates the importance of recognizing the atypical features of PRES, particularly when the presentation correlates with the diagnosis, but the MRI demonstrates an unusual location. While it is still important and necessary to test and rule out other etiologies, PRES management and treatment can occur concomitantly.

## CONFLICT OF INTEREST

The authors declare that no conflicts of interest.

## AUTHOR CONTRIBUTION

WK: wrote the abstract, introduction, and discussion; edited; and submitted the paper. Dr KI: wrote the case presentation. Dr AL: contributed to the case presentation and aided in editing the paper.

## ETHICAL APPROVAL

The authors declare that the paper was conducted in an ethical manner.
